# Data-driven symptom dimensions reveal familial patterns in bipolar disorder

**DOI:** 10.1016/j.jad.2025.121060

**Published:** 2025-12-27

**Authors:** Katie Scott, Claire O’Donovan, Sandra Meier, Barbara Pavlova, Dean F. MacKinnon, James B. Potash, Thomas G. Schulze, Jennifer Judy, Peter P. Zandi, the NIMH Genetics Initiative Bipolar Disorder Consortium, Paul Grof, Francis J. McMahon, Abraham Nunes, Martin Alda

**Affiliations:** a Department of Psychiatry, Dalhousie University, Halifax, NS, Canada; b Nova Scotia Health, Halifax, NS, Canada; c Department of Psychiatry and Behavioral Sciences, Johns Hopkins University, Baltimore, MD, USA; d Department of Psychiatry and Behavioral Sciences, Norton College of Medicine, SUNY Upstate Medical University, Syracuse, NY, USA; e Institute of Psychiatric Phenomics and Genomics, University Hospital, LMU Munich, Munich, Germany; f Mood Disorders Centre of Ottawa, Ottawa, ON, Canada; g Department of Psychiatry, University of Toronto, Toronto, ON, Canada; h National Institute of Mental Health, Bethesda, MD, USA; i Faculty of Computer Science, Dalhousie University, Halifax, NS, Canada; j National Institute of Mental Health, Klecany, Czech Republic

**Keywords:** Bipolar disorder, Phenotype, Heterogeneity, Dimensional, Family study, Principal component analysis

## Abstract

**Background:**

Bipolar disorder (BD) is a highly heritable psychiatric illness whose clinical and genetic heterogeneity complicates efforts to identify biologically-relevant subtypes. Traditional categorical approaches often fail to capture the multidimensional nature of BD symptomatology. This study aimed to evaluate whether data-driven dimensions show familial aggregation, suggesting potential genetic underpinnings.

**Methods:**

Using two independent cohorts: a primary sample from Halifax (*N* = 368) and a replication sample from the NIMH Genetics Initiative Bipolar Disorder Consortium (*N* = 1356), latent dimensions were derived from 21 clinical variables with principal component analysis (PCA). The similarity of relatives in the PCA-derived space was quantified and compared to their similarity with unrelated BD subjects. Mixed-effects models assessed whether familial similarity on latent dimensions increased with degree of relatedness.

**Results:**

Across both cohorts, the first two principal components (PCs; i.e., mood episode frequency and age of illness onset) were consistent. Overall clinical phenotype was more similar among relatives than among unrelated cases (Halifax: β = 0.316, *p* = 0.025; NIMH: β = 0.406, *p* < 0.001; Combined: β = 0.388, *p* < 0.001). PC 2 (onset) showed significant familial similarity in both cohorts, and PC 1 (episode frequency) showed similarity in the NIMH sample.

**Conclusions:**

These findings suggest that latent clinical dimensions, especially those reflecting mood episode recurrence and age of onset, aggregate within families and may reflect underlying genetic liability in BD. Dimensional, data-driven phenotypes could provide more genetically informative traits than traditional diagnostic subtypes and offer promising targets for future genetic and neurobiological research.

## 1. Introduction

Bipolar disorder (BD) is a serious psychiatric illness characterized by episodic mood disturbances ranging from elevated states of mania or hypomania to major depression. It is among the most heritable psychiatric conditions, with older twin studies estimating heritability close to 80 % (Edvardsen et al., 2008) and more recent estimates around 60 % (Johansson et al., 2019), underscoring a substantial genetic contribution to its etiology. Despite this strong genetic basis, the clinical expression of BD is considerably heterogeneous. Individuals with BD differ widely in symptom presentation, including the presence or absence of psychotic features, age at onset, episode frequency, and predominant mood polarity. This heterogeneity complicates both research and clinical care, contributing to varied study results and delays in accurate diagnosis and adequate treatment (Bauer et al., 2018). Even within the currently recognized diagnostic subtypes, bipolar I (BD-I) and bipolar II (BD-II) disorders, there is marked phenotypic variability (Nunes et al., 2022).

Our previous work using cluster analysis on detailed clinical data suggested that data-driven phenotypic profiles did not distinguish between diagnostic subtypes of BD, though they did significantly differ in lithium treatment response (Scott et al., 2024). These findings imply that defining subgroups of BD based on symptom profiles may offer greater insight into the underlying biology and treatment responsiveness of the disorder. In line with this, clinical features are consistently reported as key predictors of treatment response, underscoring their clinical relevance (Hui et al., 2019; Scott et al., 2025). However, prior efforts to define phenotypic subtypes have often relied on categorical classifications that fail to capture the multidimensional nature of BD. The complexity of the disorder is reflected by its polygenicity.

While the overall genetic contributions to BD are well established, it remains unclear which features of the clinical presentation are genetically influenced. Some aspects may be environmentally driven (Nunes et al., 2022). Still, examining familial patterns of symptom expression offers a valuable first step in identifying candidate dimensions that may be heritable. Although such designs cannot separate genetic and shared environmental influences, they can highlight traits that aggregate in families and may therefore warrant future genetic investigation.

The present study examined the similarity of clinical presentation among individuals with BD and their relatives using a dimensional, data-driven approach. We employed principal component analysis (PCA) to extract latent dimensions from clinical data, representing patterns of symptom presentation. We then quantified phenotypic similarity between probands and their relatives, as well as unrelated individuals within the PCA-derived space. This approach enabled us to evaluate overall familial similarity in the symptom dimensions while accounting for the complex interrelations among clinical variables.

We tested our hypotheses using two independent datasets: a primary sample from our Halifax site and a larger, external replication sample from the National Institute of Mental Health (NIMH) Genetics Initiative Bipolar Disorder Consortium. We hypothesized that probands and their relatives would be more similar than probands and unrelated individuals. Our secondary hypothesis was that the similarity would be greater among more closely related individuals, such as first-degree relatives (FDRs), compared to second-degree relatives (SDRs) or unrelated subjects.

## 2. Materials & methods

### 2.1. Samples

#### 2.1.1. Primary sample (Halifax cohort)

The primary sample included adult patients with BD (type I or II) and their affected relatives (FDR or SDR), drawn from longitudinal studies conducted at three sites: the Mood Disorders Program at Nova Scotia Health (including participants from the Genetics of Bipolar Disorder Family Study and unrelated subjects from the Maritime Bipolar Registry), the Mood Disorders Centre of Ottawa, and the former Hamilton Psychiatric Hospital. Recruitment and diagnostic procedures have been previously described (Nunes et al., 2020). Semi-structured diagnostic interviews (Schedule for Affective Disorders and Schizophrenia Lifetime version [(Endicott and Spitzer, 1978)], SADS-L; Structured Clinical Interview for DSM [(Kübler, 2013)], SCID) were conducted by clinicians and trained research staff. Additional information was collected during interviews such as demographics, comorbid conditions (if not included in the diagnostic interview), suicide behaviours, and detailed clinical features of BD. With consent, medical records were used as corroborating information. All participants were diagnosed according to DSM-IV criteria after consensus from psychiatrists at case presentation meetings. For consistency, we refer to this sample as the *Halifax* cohort.

#### 2.1.2. Replication sample (NIMH cohort)

The second sample was drawn from the NIMH Genetics Initiative Bipolar Disorder Consortium, hereafter referred to as the *NIMH* cohort (NCT00001174). This multisite study recruited participants and conducted assessments according to standardized protocols that have been described in detail elsewhere (Potash et al., 2007). Each individual was assessed using the Diagnostic Interview for Genetic Studies (DIGS; versions 1.0, 2.0, or 3.0; (Nurnberger et al., 1994), and diagnoses were based on DSM-III-R or DSM-IV criteria. Additional information from medical records and family informants was incorporated to support diagnostic decisions. Similarly, included subjects were adults diagnosed with BD-I or BD-II and at least one FDR or SDR relative with either diagnosis.

Across both samples, data were available for 21 variables, and only variables with less than 20 % missing data were used (see Table S1 for percent missing per variable). Briefly, the information included demographics (age, sex, marital status, employment status), diagnostic subtype, age of onset, annual episode frequency (i.e., mean episodes per year), predominant polarity, onset episode polarity, history of psychosis, history of suicide attempt, comorbid conditions (anxiety disorder, obsessive-compulsive disorder [OCD], substance use disorder, and attention-deficit hyperactivity disorder or learning disability [ADHD/LD]). Learning disabilities included intellectual disabilities and specific learning disabilities (e.g., dyslexia, dyscalculia), and diagnoses were established from medical records and prior diagnoses and/or treatment history. Additionally, all participants had a family-level identifier (family ID) and degree of relatedness to the proband. The degree of relatedness, or relatedness, was defined as the coefficient of relationship, which ranges from 0 to 1 and corresponds to the average proportion of shared genes between the proband and another individual (i.e., 1 for probands/self-comparisons, 0.5 for FDRs, 0.25 for SDRs, 0 for unrelated subjects). Detailed variable definitions are provided in Table S2.

### 2.2. Analysis

All analyses were conducted in R (version 4.4.1) using the “mice” (van Buuren and Groothuis-Oudshoorn, 2011), “PCAmixdata” (Chavent et al., 2014), “lme4” (Bates et al., 2015), and “lsa” packages (Wild, 2007). The analyses were repeated three times with minor adjustments in the randomized control pairing process, depending on the sample used (i.e., Halifax cohort, NIMH cohort, or combined).

#### 2.2.1. Multiple imputation

Multiple imputation by chained equations (MICE) was used to address missing data. The variable with the most missing data was ADHD/LD in the Halifax cohort (9.8 %) and polarity at onset in the NIMH cohort (18.9 %; Table S1). Each cohort was imputed separately, generating ten complete datasets per site. Continuous variables were imputed using predictive mean matching, and categorical variables were imputed using logistic or polytomous regression, depending on the number of categories. With the exception of the sample characteristics, all reported results reflect pooled estimates across imputations.

#### 2.2.2. Randomized control pairings

For the Halifax sample, a pool of 193 independent BD “controls” (i.e., individuals with BD that are unrelated to the family sample) was used to generate randomized pairings with probands. Randomized pairings were repeated for a set number of iterations, wherein one unrelated control was randomly assigned to each proband’s family ID for each iteration (Fig. S1).

Because the NIMH sample lacked a separate control group, we randomly selected 20 % of NIMH families to serve as a control pool, and used the remaining 80 % for the main analyses. To avoid related controls, only one subject per family ID was permitted per iteration from the control pool. For each iteration, all unrelated controls were randomly assigned a proband’s family ID (Fig. S1).

In the combined sample analysis, control selection and pairing followed a hybrid version of the above procedures, using the controls from both cohorts as the overall control pool, and assigning one random proband family ID per each unrelated BD control (Fig. S1).

To determine the number of repetitions required, we plotted similarity estimates of the controls and probands across the total number of iterations, ranging from 50 to 500 in increments of 50, and selected the point at which variability stabilized. This yielded 300 repetitions for the Halifax cohort, 100 for the NIMH cohort, and 100 for the combined sample.

#### 2.2.3. Principal component analysis for mixed data

Principal Component Analysis (PCA) for mixed data was conducted, which accommodates both categorical and continuous variables. Separate PCAs were performed for each of the three datasets (Halifax, NIMH, and combined), with component scores derived for each individual. Pooled component loadings were obtained across imputations using the average covariance matrix method (Van Ginkel and Kroonenberg, 2014), and varimax rotation was applied after pooling. Varimax rotation is an orthogonal rotation technique that was employed to simplify the component structure by maximizing the loadings of clinical variables, resulting in components with clearer interpretability and preserved independence. Components with eigenvalues >1 were used in the subsequent analyses for each sample.

#### 2.2.4. Familial similarity

In all three samples, we measured overall similarity of symptom dimensions by computing the cosine similarity between the principal component (PC; eigenvalue >1) vectors of each proband-relative or proband-control pair as it measures the angle between multidimensional vectors. Linear mixed-effects models were then used to predict cosine similarity as a function of relatedness, with family ID as a random effect, to account for potential within-family variance and the varying number of relatives per family. Cohort was included as a covariate when modeling the combined sample. As a sensitivity analysis, we conducted the same process using Euclidean distance to measure proximity in place of cosine similarity. To further evaluate the familiality of individual PCs, we compared the single values representing component coordinates for each subject. Because cosine similarity requires vector inputs, we employed Euclidean distance to assess similarity at the component level. We modeled Euclidean distance between probands and other groups (relatives or unrelated controls) as a function of relatedness, again using mixed-effects models with family ID as a random effect, and cohort as a covariate for the combined sample.

We allowed for the number of outcomes to vary per sample (Halifax, NIMH, and combined) depending on the components with eigenvalues >1 derived from PCA. The Benjamini-Hochberg method for false-discovery rate was used to correct for multiple comparisons of fixed effects (i.e., relatedness and cohort) across all samples and significance was defined as corrected-*p* < 0.05.

## 3. Results

### 3.1. Sample characteristics

Descriptive statistics for each sample are presented in Table 1. The Halifax sample (*N* = 368) included 71 probands, 83 FDRs, 21 SDRs, and 193 unrelated BD cases. The NIMH sample (*N* = 1356) included 442 probands, 654 FDRs, and 104 SDRs, as well as 156 BD cases used as the control pool, from 111 independent families. The self-reported ethnic background of participants in either sample was primarily of European ancestry, with 91.8 % reporting as such in the Halifax cohort, and 88.8 % in the NIMH cohort.

There were a few notable differences in the distributions of demographic and clinical characteristics across the two cohorts. A larger proportion of subjects in the NIMH cohort were diagnosed with BD-I (83.8 %) compared to the Halifax sample (63.6 %). Consistent with this, the NIMH sample had an earlier age of illness onset (mean: 20.6 years), and earlier ages of onset for the first depressive (20.3) and manic or hypomanic (24.6) episodes, in contrast to the Halifax cohort (age of onset = 24.1; depression onset = 22.6; mania onset = 28.4). Further, there was a higher prevalence of comorbid substance use disorder in the NIMH sample (43.1 %) than in the Halifax sample (31.0 %). On the other hand, the Halifax cohort had higher rates of the three other comorbidities (anxiety disorder = 44.0 %; OCD = 9.7 %; ADHD/LD = 9.6 %) compared to NIMH (anxiety disorder = 25.9 %; OCD = 5.3 %; ADHD/LD = 6.8 %). Lastly, more participants in the Halifax group were unable to work and received disability benefits due to their psychiatric illness (28.1 %) than those in the NIMH sample (13.8 %), which may reflect differences in the Canadian and American healthcare and pension systems.

### 3.2. Principal component analysis for mixed data

**Fig. 1.**
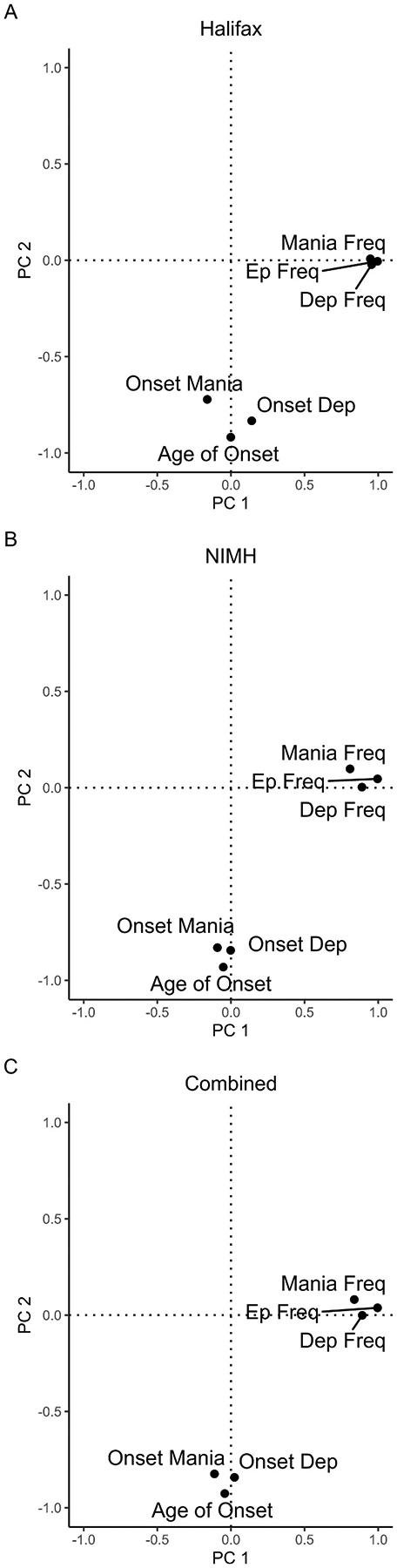
Plots of principal components 1 and 2 by sample. Only variables with squared loadings >0.1 after varimax rotation are shown. *Abbreviations*: Dep, depression; Ep, episode; Freq, frequency; PC, principal component.

PCA for mixed data was performed separately for the Halifax, NIMH, and combined datasets, and principal components with eigenvalues greater than 1 were retained for the remaining analyses. The first three components, explaining 65.9 % of variance, were selected for the Halifax dataset, and both the NIMH and combined analyses resulted in two components, explaining 52.1 % of the variance in those datasets (Fig. S2). The varimax-rotated squared loadings of all variables per sample are reported in Table 2, and the unrotated squared loadings are provided in the supplementary materials (Table S3). In all three samples, the first two PCs captured the same variables (Fig. 1). See Fig. S3 for plots of the remaining PC comparisons from the Halifax cohort.

PC 1 loaded strongly on variables reflecting episode frequency; specifically, the average number of depressive, manic/hypomanic, and total mood episodes per year of illness. Squared loadings across the three cohorts ranged from 0.793 to 0.913 for depressive episodes per year, 0.655–0.898 for manic/hypomanic episodes per year, and 0.991–0.992 for total episodes per year.

PC 2 primarily reflected variation in age of onset, with strong loadings from age of illness onset, age at first depressive episode, and age at first manic/hypomanic episode. Squared loadings across cohorts ranged from 0.843 to 0.867 for age at overall onset, 0.694–0.712 for age at first depressive episode, and 0.521–0.689 for age at first manic/hypomanic episode.

Only the Halifax cohort retained a third component with an eigenvalue greater than 1 (Table 2). In this sample, PC 3 loaded on diagnostic subtype (BD-I or BD-II), predominant polarity (manic/hypomanic, depressive, or balanced), first episode polarity (manic/hypomanic or depressive), and age at onset of the first manic/hypomanic episode. Squared loadings for PC 3 in the Halifax cohort were 0.229 for age at first mania, 0.169 for diagnosis, 0.228 for predominant polarity, and 0.321 for first episode polarity. The directionality of variable coordinates further clarifies the nature of this component: BD-I was associated with a coordinate of 0.336, and BD-II with −0.577; manic/hypomanic predominant polarity loaded at 0.555, depressive predominant polarity at −0.598, and balanced polarity at 0.128; first episode manic polarity loaded at 0.616 and first episode depressive polarity at −0.527. The age of the first manic/hypomanic episode had a coordinate of −0.478. More positive scores on this component were thus associated with BD-I diagnosis, manic first episode polarity, manic predominant polarity, and later onset of mania, while more negative scores were associated with BD-II diagnosis, depressive first episode and predominant polarity, and earlier onset of manic symptoms. These findings suggest that PC 3 captures a broader dimension related to polarity and illness trajectory. Variable coordinates for components in each sample are available in the supplementary materials (Table S4).

### 3.3. Overall familial similarity

**Fig. 2.**
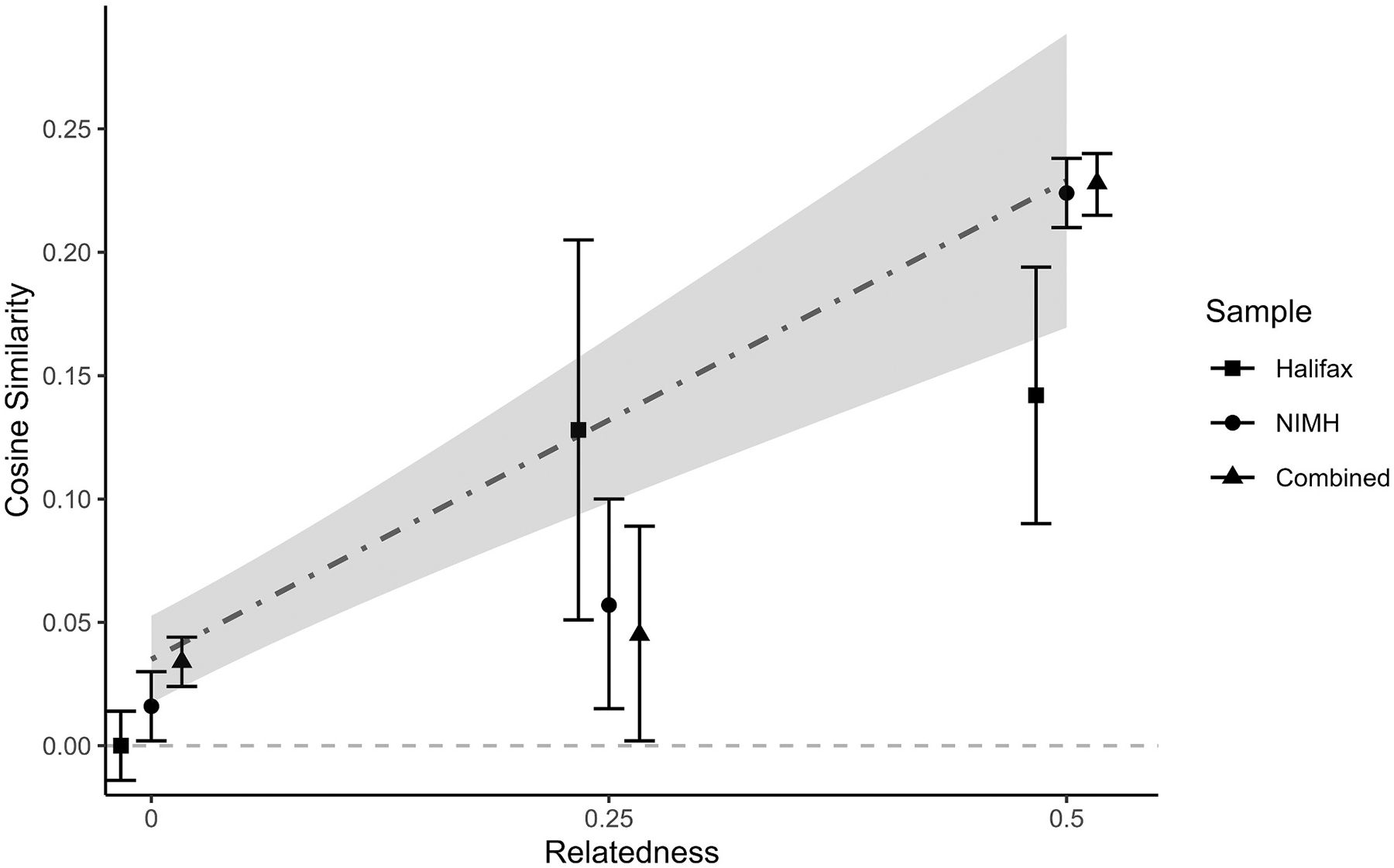
Cosine similarity by relatedness. Pooled results of overall cosine similarity in each sample, stratified by relatedness. Error bars are 95 % confidence intervals. Fixed-effect regression line plotted for the combined sample with the shaded area representing the 95 % confidence interval for the effect mean.

The cosine similarity by relatedness for all samples is shown in Fig. 2. In the Halifax sample, cosine similarity between individuals increased significantly with relatedness (β = 0.316, SE = 0.141, corrected-*p* = 0.025), and the NIMH cohort showed consistent findings (β =0.406, SE = 0.063, corrected-*p* < 0.001). In the combined sample, the effect of relatedness remained significant (β = 0.388, SE = 0.058, corrected-*p* < 0.001), and there was no effect of cohort (β = −0.005, SE = 0.013, corrected-p = 0.755), suggesting consistent overall familial similarity across cohorts (Table 3). The same models were analyzed using Euclidean distance to measure similarity/dissimilarity, and the results remained consistent across all samples (Table S5).

### 3.4. Familial similarity of individual principal components

To assess which specific dimensions showed familial similarity, we analyzed the Euclidean distance for each principal component separately (Table 4).

There was no significant effect of relatedness on the Euclidean distance for PC 1 in the Halifax cohort (β = −0.103, corrected-*p* = 0.722). In contrast, PC 1 in the NIMH and combined samples both showed a significant effect (NIMH: β = −0.261, corrected-*p* = 0.007; Combined: β = −0.207, corrected-*p* = 0.025), where the more closely related individuals were closer in the PCA-derived space. Additionally, the cohort did not have a significant effect in the combined model (β = 0.006, corrected-*p* = 0.866).

PC 2 showed a significant relationship with relatedness in all three samples (Halifax: β = −0.356, corrected-*p* = 0.047; NIMH: β = −0.194, corrected-*p* = 0.004; Combined: β = −0.219, corrected-*p* < 0.001). Notably, there was a cohort effect in the combined model (β = −0.046, corrected-*p* = 0.002), suggesting that subjects in the NIMH cohort were generally more similar to each other. However, there was no interaction effect of relatedness and cohort when a model was rerun with the interaction term included (β = 0.179, *p* = 0.339), whereas the fixed effects remained significant (Relatedness: β = −0.370, *p* = 0.035; Cohort: β = −0.046, *p* < 0.001), indicating that the effect of relatedness on Euclidean distance did not differ by cohort.

Finally, PC 3 in the Halifax sample showed only a trend, prior to correction, of closer relatives having less distance than more distantly related and unrelated individuals (β = −0.299, *p* = 0.095, corrected-*p* = 0.123).

### 3.5. Testing familiality of individual variables

Upon observing the higher squared loadings of total episode frequency and age of illness onset for components 1 and 2, respectively, we ran additional mixed-effect regressions for the individual continuous variables (i.e., frequency of total episodes, frequency of depressions, frequency of manias, age of onset, onset of depression, onset of mania) to examine whether the familiality of the PCs were driven by certain features. The mixed-effects models specifically tested whether relatedness predicted the absolute difference for each standardized variable between relatives or controls and the probands, while controlling for the random effect of family membership. The Benjamini-Hochberg method was again used to adjust for false-discovery rate. Briefly, none of the variables tested were significant in the Halifax cohort after correction. In contrast, the NIMH cohort showed age of onset (β = −0.24, corrected-*p* ≤ 0.001) and onset of mania (β = −0.26, corrected-*p* ≤ 0.001) were familial in that higher degrees of relatedness had smaller absolute differences with probands, whereas onset of depression showed only a trend towards familiality (β = −0.11, corrected-*p* = 0.098). As for the frequency variables, familiality was shown in the frequency of depressive episodes (β = −0.36, corrected-*p* = 0.001), while the frequency of total episodes was nominally significant (β = −0.30, corrected-*p* = 0.098), and frequency of mania did not exhibit familiality (β = −0.16, corrected-*p* = 0.417). The full results of the mixed-effect regressions are reported in Table S6.

## 4. Discussion

This study aimed to evaluate whether symptom patterns in BD show familial aggregation when transformed into a lower-dimensional space using PCA. Across two independent cohorts, we found that symptom dimensions, based on the overall cosine similarity of coordinates on retained components, were more similar among related individuals than among unrelated individuals, and that this similarity increased with genetic relatedness in regression models.

The reported intercohort differences of sample characteristics appear primarily driven by the disparity in proportion of BD-II diagnosis. Specifically, the Halifax sample had a higher prevalence of BD-II diagnoses compared to the NIMH cohort and also showed greater prevalence of several comorbid conditions and an older age of illness onset. While our goal is not to classify differences solely based on diagnostic subtype, we present these distinctions in this context because prior literature suggests that at least a subgroup of individuals diagnosed with BD-II tend to exhibit more comorbid anxiety (Rihmer et al., 2001) and a later age of onset (Serafini et al., 2019), compared to those diagnosed with BD-I, though not all studies report this (Brancati et al., 2023; Mantere et al., 2006). Importantly, despite this difference in sample characteristics, the effect of familiality for overall clinical phenotype was consistent across both the Halifax and NIMH cohorts, which strengthens the validity and generalizability of our findings. This consistency further suggests that our PCA-derived components capture biologically-relevant dimensions of the BD phenotype, beyond cohort-specific features or diagnostic practices.

The principal components identified were largely stable across samples. The first component, which reflected mood episode frequency, and the second component, capturing age of onset, were replicated and showed significant familial aggregation. Specifically, PC 2 (age of onset) demonstrated significant familial similarity in all three datasets, and PC 1 (episode frequency) did so in the larger NIMH and combined samples. These findings align with prior evidence that both age of onset and episode recurrence in BD are familial traits (Baldessarini et al., 2012; Fisfalen et al., 2005; Kassem et al., 2006; Schulze et al., 2006; Scott et al., 2023). Furthermore, our findings are consistent with other studies seeking to identify data-driven phenotypic dimensions of BD. Baek et al. (2019) identified 6 dimensions using clinical data related to the course of illness, mood symptoms, and comorbidities in a Korean sample. Of the six dimensions derived, one they labeled as “cyclicity” (Baek et al., 2019), which is similar to our episode frequency dimension. However, PC 1 may represent a less stable familial trait, as the frequency of episodes is expected to decrease with adequate treatment. In particular, the Halifax cohort was obtained from longitudinal studies, where many participants were active patients at the clinic sites. Consequently, ongoing data collection in this cohort may obscure the familial effect for PC 1 that was observed in the NIMH sample.

The third component, unique to the Halifax cohort, reflected a trajectory-related dimension involving diagnostic subtype, polarity of illness onset, onset of mania, and predominant polarity. The absence of this component in the larger NIMH cohort may be due to the proportionately smaller number of individuals with BD-II compared to the Halifax cohort, since BD-II is often associated with a depressive episode at onset and a predominantly depressive polarity (Brancati et al., 2023).

When examining cosine similarity by degree of relatedness for all samples, probands and their FDRs were significantly more similar than the probands and controls. In contrast, similarity between probands and SDRs did not significantly differ from that between probands and controls in the NIMH cohort and the combined sample, suggesting that the observed familial aggregation was primarily driven by the FDRs in those groups. In the Halifax cohort, both FDRs and SDRs were significantly different from controls, but the similarity of either degree of relative to probands did not differentiate. One possible explanation for this discrepancy is statistical power: the Halifax sample was smaller overall and contained a higher proportion of SDRs compared to NIMH, leaving estimates for this group especially underpowered and associated with wide confidence intervals. Beyond issues of precision, genetic and environmental contributions to familial resemblance may also partition differently across cohorts. While genetic sharing between FDRs and SDRs should be comparable across samples, environmental influences may vary. For instance, in the Halifax cohort, SDRs may have been raised in more similar contexts to probands than was the case for SDRs recruited across multiple sites in the NIMH sample. Families may also be more communicative and open about mental illness in one cohort compared to the other, which could amplify familial, non-genetic effects. For example, if it is known that the proband experiences an early age of onset, other family members may be prompted to seek clinical assessment or diagnosis sooner than they otherwise would. This could lead to earlier recognition of illness among extended relatives, including SDRs, and result in a reported age of onset that is more similar to probands. Earlier recognition may also reduce recall bias and other limitations associated with retrospective determination of onset, which could make differences between degrees of relatives more difficult to detect. Broader systemic factors may also contribute. In Canada, universal health coverage could lead to more consistent evaluation and treatment of relatives across families, while in the NIMH sample drawn from diverse U.S. sites, variability in access and care due to financial or systemic factors may have introduced greater heterogeneity. These contextual differences could help explain the lack of differentiation in similarity by degree of relatedness in the Halifax cohort. As a whole, these findings support the hypothesis that certain aspects of the BD clinical phenotype may reflect underlying familial, and possibly genetic, contributions, even when assessed using dimensional rather than categorical frameworks.

Our findings offer several noteworthy implications. First, they support the value of dimensional, data-driven approaches for characterizing phenotypic variability in BD. Traditional categorical subtypes like BD-I and BD-II may obscure meaningful underlying variation. By contrast, latent dimensions such as episode frequency and age at onset appear to represent biologically and genetically relevant aspects of the illness. These components could serve as more informative phenotypes for future genetic studies, particularly in efforts to identify genotypephenotype correlations. Indeed, Baek and colleagues investigated the polygenic basis of the dimensions identified in their prior study and found cyclicity was negatively associated with the polygenic score for schizophrenia (Baek et al., 2024). However, it is intriguing that the two dimensions we identified are not particularly unique to BD but instead represent broadly shared facets across multiple psychiatric conditions (Meuret et al., 2006; Musliner et al., 2016; Nowrouzi et al., 2015; Zisook et al., 2004). This raises an important question regarding which components truly capture the distinct qualitative profile of BD, as opposed to aspects overlapping with other disorders such as schizophrenia. Our findings suggest a possible indication of this phenotypic signature in the third component identified in the Halifax cohort, which appears to reflect features more specific to BD. Future work comparing BD with other psychiatric populations will be critical to delineate disorder-specific latent dimensions from those that are transdiagnostic, thereby refining phenotype definitions and improving specificity of both genetic and clinical investigations.

Second, the observed familial similarity in PCA-derived symptom dimensions highlights a promising pathway for addressing the clinical heterogeneity of BD using genetic analytical methods. Rather than seeking one-to-one relationships between specific genes and diagnostic subtypes, future work may benefit from focusing on transdiagnostic or dimensional traits that aggregate within families and reflect shared genetic underpinnings. Such efforts could complement genomic findings by providing richer phenotypic targets for analysis and reducing noise associated with diagnostic categories.

Despite these strengths, several limitations should be considered. First, we employed MICE to impute missing data which assumes information is missing at random, while non-random missingness is common in real world datasets. To test this assumption, we calculated the correlation between the proportion of missingness in variables and the squared loadings of PCs. Variable missingness from the Halifax cohort did not show any significant correlations with squared loadings, while there was one notable association in the NIMH data. Specifically, loadings for PC 3 in the NIMH cohort had a significant positive correlation with the proportion of missing data, even after applying the Benjamini-Hochberg adjustment (*r* = 0.81, CI 95 %: [0.52, 0.94], corrected-*p* = 0.003; Table S7). Given this, the third dimension in the NIMH cohort may have been influenced by a non-random pattern of missingness, and therefore could have led to the discrepancy of the retained components between our samples. Second, while PCA allows for dimensionality reduction, the method is sensitive to sample-specific variance, and this may be another explanation as to why the third component observed in the Halifax sample did not generalize to the NIMH dataset. Third, although our use of cosine similarity provides a multivariate assessment of resemblance, it does not identify the specific mechanisms, genetic or environmental, responsible for the observed familial aggregation. Also, both datasets were of predominantly European ancestry, according to self-report data. This may limit the generalizability of our findings as there is prior evidence of differential clinical presentations in BD for certain ethnic groups (Li et al., 2023). Lastly, the control groups differed between the two datasets: the Halifax cohort included independently recruited unrelated controls, while the NIMH sample used intracohort randomization to form a control group. Although the similarity trends remained consistent, these differences may have influenced the magnitude of similarity scores. For the Halifax cohort, controls were recruited for a BD registry rather than a family study, and may have been less likely to have a family history of BD. This could make them more distinct from the family-based cases, especially if familial and non-familial BD differ in phenotype, potentially amplifying the observed familial effect. The significant effect of cohort in the combined sample for PC 2, indicating that subjects in the NIMH cohort were more similar to one another compared to the Halifax cohort, may further support this notion.

In conclusion, this study demonstrates that latent symptom dimensions in BD, particularly those related to mood episode frequency and illness onset, are more similar among genetically related individuals, even after reducing the phenotype to a multivariate, dimensional space. These findings underscore the potential utility of data-driven symptom modeling in capturing clinically and genetically meaningful variation in BD. Next steps could include testing the replicability of the third “polarity” dimension in a well-characterized independent sample. Future studies should explore how these phenotypic dimensions relate to specific genetic markers and whether they can improve prediction of illness course or treatment response.

## Supplementary Material

1

## Figures and Tables

**Table 1:** Sample Characteristics.

Variable		Halifax *N* = 368	NIMH *N* = 1356	Combined *N* = 1724
Age		44.4 (13.5)	42.5 (13.3)	42.9 (13.4)
Sex (%)	Male	135 (36.7)	514 (37.9)	649 (37.6)
	Female	233 (63.3)	842 (62.1)	1075 (62.4)
Relatedness (%)	1 [Proband]	71 (19.3)	442 (32.6)	513 (29.8)
	0.5 [FDR]	83 (22.6)	654 (48.2)	737 (42.7)
	0.25 [SDR]	21 (5.7)	104 (7.7)	125 (7.3)
	0 [Control]	193 (52.4)	156 (11.5)	349 (20.2)
Marital Status (%)	Married	197 (53.8)	674 (49.8)	871 (50.7)
	Single	169 (46.2)	679 (50.2)	848 (49.3)
Employment Status (%)	Employed	192 (53.9)	931 (68.7)	1123 (65.6)
	Unemployed	64 (18.0)	238 (17.6)	302 (17.6)
	Disability	100 (28.1)	187 (13.8)	287 (16.8)
Diagnosis (%)	BD-I	234 (63.6)	1137 (83.8)	1371 (79.5)
	BD-II	134 (36.4)	219 (16.2)	353 (20.5)
Age of Onset		24.1 (9.4)	0.6 (9.4)	21.3 (9.5)
Depression Onset		22.6 (11.5)	20.3 (11.1)	20.8 (11.2)
Mania Onset		28.4 (11.1)	24.6 (10.6)	25.5 (10.8)
Polarity at Onset (%)	Depressive	229 (62.9)	699 (63.5)	928 (63.4)
	Manic	135 (37.1)	401 (36.5)	536 (36.6)
Episode Frequency		0.9 (1.5)	1.1 (2.5)	1.1 (2.3)
Depression Frequency		0.4 (0.6)	0.6 (1.4)	0.6 (1.3)
Mania Frequency		0.5 (1.1)	0.7 (1.7)	0.7 (1.6)
Predominant Polarity (%)	Balanced	153 (42.0)	423 (32.7)	576 (34.7)
	Depressive	127 (34.9)	457 (35.3)	584 (35.2)
	Manic	84 (23.1)	415 (32.0)	499 (30.1)
Psychosis (%)	No	164 (46.7)	678 (52.4)	842 (51.2)
	Yes	187 (53.3)	616 (47.6)	803 (48.8)
Suicide Attempt (%)	No	221 (65.2)	870 (65.0)	1091 (65.0)
	Yes	118 (34.8)	469 (35.0)	587 (35.0)
Anxiety Disorder (%)	No	196 (56.0)	1005 (74.1)	1201 (70.4)
	Yes	154 (44.0)	351 (25.9)	505 (29.6)
OCD (%)	No	317 (90.3)	1284 (94.7)	1601 (93.8)
	Yes	34 (9.7)	72 (5.3)	106 (6.2)
Substance Use Disorder (%)	No	243 (69.0)	772 (56.9)	1015 (59.4)
	Yes	109 (31.0)	584 (43.1)	693 (40.6)
ADHD/LD (%)	No	300 (90.4)	1263 (93.2)	1563 (92.6)
	Yes	32 (9.6)	92 (6.8)	124 (7.4)

*Note*. Continuous variables reported as mean (standard deviation) and categorical variables as N (percent). Episode frequency variables are the mean number of episodes (total mood episodes, major depressive episodes, or (hypo)manic episodes) per year of illness. *Abbreviations:* ADHD/LD, attention-deficit hyperactivity disorder and/or learning disability; BD-I, bipolar I disorder; BD-II, bipolar II disorder; FDR, first-degree relatives; OCD, obsessive-compulsive disorder; SDR, second-degree relatives.

**Table 2:** Pooled Squared Loadings per Cohort.

Variable	Halifax			NIMH		Combined	
	PC 1 (31.2 %)	PC 2 (23.4 %)	PC 3 (11.3 %)	PC 1 (29.2 %)	PC 2 (22.9 %)	PC 1 (28.9 %)	PC 2 (23.2 %)
Age of onset	0.000	**0.843**	0.028	0.003	**0.867**	0.002	**0.857**
Depression onset	0.020	**0.694**	0.003	0.000	**0.712**	0.001	**0.709**
Mania onset	0.026	**0.521**	**0.229**	0.008	**0.689**	0.012	**0.679**
Episode frequency	**0.992**	0.000	0.002	**0.991**	0.002	**0.992**	0.001
Depression frequency	**0.913**	0.001	0.013	**0.793**	0.000	**0.796**	0.000
Mania frequency	**0.898**	0.000	0.025	**0.655**	0.010	**0.703**	0.007
Diagnosis	0.002	0.001	**0.169**	0.000	0.000	0.000	0.000
Predominant polarity	0.007	0.016	**0.228**	0.003	0.000	0.003	0.001
Polarity at onset	0.011	0.000	**0.321**	0.003	0.001	0.000	0.000
Psychosis	0.003	0.003	0.069	0.001	0.011	0.001	0.011
Suicide attempt	0.011	0.037	0.013	0.010	0.028	0.009	0.029
Anxiety disorder	0.024	0.053	0.016	0.009	0.009	0.011	0.012
OCD	0.000	0.000	0.000	0.001	0.003	0.000	0.002
Substance use disorder	0.011	0.002	0.036	0.002	0.009	0.003	0.007
ADHD/LD	0.001	0.007	0.000	0.000	0.001	0.000	0.001

Squared loadings of principal components (eigenvalue >1) after varimax rotation, pooled across all imputed datasets per sample. Values >0.1 in bold. Squared loadings of variables are squared correlations and correlation ratios between the variable and principal component, for quantitative and qualitative variables, respectively. Percent of explained variance is given under each component. *Abbreviations:* ADHD/LD, attention-deficit hyperactivity disorder and/or learning disability; OCD, obsessive-compulsive disorder; PC, principal component.

**Table 3:** Mixed Effects Model Results of Similarity by Sample.

	Model: Cosine Similarity ~ Relatedness + (1 | Family ID)						
Sample	Term	*β*	SE	*β*/SE	*df*	*p*	Corrected-*p*
Halifax	Intercept	0.003	0.012	0.208	286	0.836	–
	Relatedness	0.316	0.141	2.245	609	0.025	**0.041**
NIMH	Intercept	0.019	0.010	1.927	48	0.060	–
	Relatedness	0.406	0.063	6.410	633	< 0.001	< **0.001**
	Model: Cosine Similarity ~ Relatedness + Cohort + (1 | Family ID)						
Combined	Intercept	0.035	0.009	3.786	82	< 0.001	–
	Relatedness	0.388	0.058	6.645	745	< 0.001	< **0.001**
	Cohort: NIMH	−0.005	0.013	−0.393	31	0.697	0.755

Fixed effect significance of corrected-*p* < 0.05 in bold. Corrected-*p* used Benjamini-Hochberg method for 13 comparisons. *Abbreviations*: PC, principal component.

**Table 4:** Similarity Results for Individual Principal Components by Sample.

Halifax	Model: Euclidean Distance ~ Relatedness + (1 | Family ID)						
Component	Term	*β*	SE	*β*/SE	*df*	*p*	Corrected-*p*
PC 1	Intercept	0.865	0.102	8.516	2768	< 0.001	–
	Relatedness	−0.103	0.203	−0.510	891	0.611	0.722
PC 2	Intercept	1.082	0.051	21.055	403	< 0.001	–
	Relatedness	−0.356	0.166	−2.143	1112	0.032	**0.047**
PC 3	Intercept	1.260	0.059	21.513	73	< 0.001	–
	Relatedness	−0.299	0.179	−1.673	1136	0.095	0.123
NIMH	Model: Euclidean Distance ~ Relatedness + (1 | Family ID)						
Component	Term	*β*	SE	*β*/SE	*df*	*p*	Corrected-*p*
PC 1	Intercept	0.631	0.099	6.406	11	< 0.001	–
	Relatedness	−0.261	0.086	−3.039	108	0.003	**0.007**
PC 2	Intercept	1.064	0.024	44.415	2086	< 0.001	–
	Relatedness	−0.194	0.061	−3.172	6554	0.002	**0.004**
Combined	Model: Euclidean Distance ~ Relatedness + Cohort + (1 | Family ID)						
Component	Term	*β*	SE	*β*/SE	*df*	*p*	Corrected-*p*
PC 1	Intercept	0.681	0.097	7.052	12	< 0.001	–
	Relatedness	−0.207	0.082	−2.531	79	0.013	**0.025**
	Cohort: NIMH	0.006	0.032	0.172	11	0.866	0.866
PC 2	Intercept	1.083	0.022	49.874	776	< 0.001	–
	Relatedness	−0.219	0.056	−3.890	8125	< 0.001	< **0.001**
	Cohort: NIMH	−0.046	0.012	−3.816	38	< 0.001	**0.002**

*Note*. Fixed effect significance of corrected-*p* < 0.05 in bold. Corrected-*p* used Benjamini-Hochberg method for 13 comparisons. *Abbreviations*: PC, principal component.

## Appendix A. Supplementary data

Supplementary data to this article can be found online at https://doi.org/10.1016/j.jad.2025.121060.
