# Supplementary material for: Data-driven symptom dimensions reveal familial patterns in bipolar disorder
Source: J Affect Disord. Author manuscript; Available in PMC 2026 Jul 8. (PMC13343236; doi:10.1016/j.jad.2025.121060)
Supplement: 1 [file NIHMS2188767-supplement-1.pdf]

Supplementary Materials

Title: Data-driven symptom dimensions reveal familial patterns in bipolar disorder
Authors: Katie Scott, Claire O'Donovan, Sandra Meier, Barbara Pavlova, Dean F MacKinnon, James B Potash, Thomas G Schulze, Jennifer Judy, Peter P Zandi, the NIMH Genetics Initiative Bipolar Disorder Consortium, Paul Grof, Francis J McMahon, Abraham Nunes, Martin Alda.

Table S1. Missing Data in Variables per Sample.

Variable	Missing Data	
	**Halifax**	**NIMH**
Family ID	0.0%	0.0%
Age	0.0%	0.0%
Sex	0.0%	0.0%
Marital Status	0.5%	0.2%
Employment Status	3.3%	0.0%
Relatedness	0.0%	0.0%
Diagnosis	0.0%	0.0%
Age of Onset	0.0%	0.0%
Depression Onset	2.4%	2.5%
Mania Onset	2.4%	6.8%
Episode Frequency	5.4%	0.0%
Depression Frequency	4.6%	11.7%
Mania Frequency	6.3%	15.9%
Polarity at Onset	1.1%	18.9%
Predominant Polarity	1.1%	4.8%
Psychosis	4.6%	4.3%
Suicide Attempt	7.9%	1.3%
Anxiety Disorder	4.9%	0.0%
OCD	4.6%	0.0%
Substance Use Disorder	4.3%	0.0%
ADHD/LD	9.8%	0.1%

Abbreviations: ADHD/LD, attention-deficit hyperactivity disorder and/or learning disability; OCD, obsessive-compulsive disorder.

Table S2. Variable Definitions.

Variable	Description
Family ID	Family identifier
Age	Age at last interview
Sex	Biological sex assigned at birth<br>Male<br>Female
Relatedness	Coefficient of relatedness; average proportion of shared genetics.<br>1: self/proband<br>0.5: first-degree relatives (e.g., parent, sibling, child)<br>0.25: second-degree relatives (e.g., aunt/uncle, niece/nephew)<br>0: unrelated controls
Marital Status	Single: Includes divorced (and not remarried), and separated.<br>Married: Includes common-law status and widowed.
Employment Status	Employed: Includes full-time or part-time, self-employed, retired, and student.<br>Unemployed: Includes receiving unemployment insurance or social assistance/welfare, and homemakers.<br>Disabled: Receiving disability pension or other disability income/benefits due to their psychiatric illness.
Diagnosis	Diagnostic subtype of bipolar disorder (BD).<br>BD-I: bipolar I disorder<br>BD-II: bipolar II disorder
Age of Onset	Age of onset for bipolar disorder.<br>Halifax: the age at which the individual met diagnostic criteria for either a major depressive episode or a hypomanic/manic episode.<br>NIMH: the age at which the individual met diagnostic criteria for either a major depressive episode or a manic episode. Hypomanic onsets were not considered, due to low reliability.
Depression Onset	Age of first major depressive episode, as defined by diagnostic criteria.
Mania Onset	Age of first hypomanic or manic episode, as defined by diagnostic criteria.
Polarity at Onset	Polarity of the episode at illness onset (i.e., index episode).<br>Depressive<br>Manic: hypomanic or manic polarity
Episode Frequency	Mean number of mood episodes per year of illness.

Depression Frequency	Mean number of major depressive episodes per year of illness.
Mania Frequency	Mean number of hypomanic and manic episodes (combined) per year of illness.
Predominant Polarity	Predominant polarity of mood episodes. Where two-thirds or more of lifetime episodes are a single polarity (i.e., depressive or manic/hypomanic), otherwise predominant polarity is considered balanced.<br>Balanced<br>Depressive<br>Manic
Psychosis	A history of psychotic features during any mood episodes.<br>Yes<br>No
Suicide Attempt	A history of one or more suicide attempts.<br>Yes<br>No
Anxiety Disorder	Meets diagnostic criteria for one or more comorbid anxiety disorders; includes any of the following: social anxiety disorder, panic disorder, or generalized anxiety disorder.<br>Yes<br>No
OCD	Meets diagnostic criteria for obsessive-compulsive disorder (OCD).<br>Yes<br>No
Substance use disorder	Meets diagnostic criteria for substance use disorder. Includes alcohol, cannabis, or other illicit substances.<br>Yes<br>No
ADHD/LD	Meets diagnostic criteria for attention-deficit hyperactivity disorder (ADHD) and/or any learning disability (LD).<br>Yes<br>No

Table S3. All Unrotated Squared Loadings per Sample

**Halifax**	**PC 1**	**PC 2**	**PC 3**	**PC 4**	**PC 5**	**PC 6**	**PC 7**	**PC 8**	**PC 9**	**PC 10**	**PC 11**	**PC 12**	**PC 13**	**PC 14**	**PC 15**
Age of Onset	0.001	0.757	0.112		0.018	0.001	0.021	0.038	0.020	0.002	0.004	0.018	0.007	0.001	
Depression Onset	0.007	0.703	0.007	0.009	0.214	0.015	0.009	0.011	0.002	0.004	0.014	0.001	0.005		
Mania Onset	0.075	0.606	0.094	0.002	0.151	0.022	0.001	0.008	0.015	0.001	0.001	0.020	0.003		
Episode Frequency	0.977	0.007	0.009	0.002	0.004										
Depression Frequency	0.854	0.016	0.057	0.005	0.002	0.004	0.001	0.006	0.011	0.002	0.025	0.005		0.010	0.003
Mania Frequency	0.920	0.002		0.014	0.005	0.002		0.006	0.011	0.002	0.020	0.007		0.007	0.002
Diagnosis	0.011	0.001	0.160	0.030	0.005	0.008	0.026	0.016	0.011	0.090	0.003	0.010	0.006		
Predominant Polarity	0.017	0.016	0.217	0.083	0.016	0.071	0.027	0.008	0.128	0.009	0.043	0.001	0.001	0.012	0.012
Polarity at Onset	0.035	0.008	0.288	0.055	0.001	0.001		0.016		0.035		0.045		0.001	0.002
Psychosis		0.011	0.064	0.183	0.094	0.053	0.044	0.004		0.028	0.003	0.008			
Suicide Attempt	0.009	0.025	0.027	0.164	0.009	0.052	0.003	0.114			0.020	0.004		0.001	
Anxiety	0.034	0.055	0.003	0.080		0.001	0.205	0.026	0.020	0.002	0.004	0.004			
OCD						0.003				0.001	0.003	0.001		0.027	0.021
Substance Use	0.017		0.032	0.032	0.022	0.199	0.001	0.022	0.006	0.024	0.027	0.004			
ADHD/LD		0.007		0.004	0.001	0.001	0.004	0.015	0.002	0.003	0.004	0.007	0.101		
**NIMH**	**PC 1**	**PC 2**	**PC 3**	**PC 4**	**PC 5**	**PC 6**	**PC 7**	**PC 8**	**PC 9**	**PC 10**	**PC 11**	**PC 12**	**PC 13**	**PC 14**	**PC 15**
Age of Onset	0.381	0.489	0.025			0.001	0.010	0.004	0.018	0.002	0.007	0.061		0.001	
Depression Onset	0.274	0.438	0.048	0.056	0.051		0.025	0.014	0.054	0.021	0.013	0.005		0.001	
Mania Onset	0.343	0.354	0.158	0.001	0.074		0.007		0.019	0.005		0.039			
Episode Frequency	0.657	0.337	0.001	0.003	0.001	0.001									
Depression Frequency	0.492	0.301	0.050	0.015	0.105	0.013		0.007	0.012		0.005				
Mania Frequency	0.485	0.180	0.088	0.007	0.186	0.013	0.003	0.007	0.023		0.009				
Diagnosis															0.017
Predominant Polarity	0.001	0.002	0.136	0.027	0.033	0.007	0.126	0.006	0.017	0.183	0.125	0.001		0.001	
Polarity at Onset	0.003		0.313	0.008	0.048		0.020	0.012	0.035	0.001	0.024	0.026		0.001	
Psychosis	0.002	0.009	0.005	0.013	0.025	0.404		0.011	0.003	0.011					
Suicide Attempt	0.032	0.005	0.005	0.147	0.020	0.012	0.070	0.158	0.033	0.002					
Anxiety	0.018			0.047	0.021	0.023	0.117	0.007	0.102	0.047			0.005		
OCD	0.003	0.001		0.003	0.001		0.006		0.002			0.003	0.071	0.030	
Substance Use	0.009	0.003	0.004	0.252		0.002	0.028	0.184	0.006	0.005					
ADHD/LD		0.001	0.002	0.003		0.001	0.001	0.001	0.003	0.003	0.002	0.002	0.036	0.057	
**Combined**	**PC 1**	**PC 2**	**PC 3**	**PC 4**	**PC 5**	**PC 6**	**PC 7**	**PC 8**	**PC 9**	**PC 10**	**PC 11**	**PC 12**	**PC 13**	**PC 14**	**PC 15**
Age of Onset	0.293	0.566	0.031	0.001	0.002	0.001	0.013		0.023	0.001	0.003	0.063	0.001	0.001	

Depression Onset	0.194	0.515	0.041	0.074	0.050		0.038		0.050	0.022	0.010	0.006			
Mania Onset	0.296	0.395	0.160	0.007	0.075		0.006	0.001	0.013	0.004	0.001	0.040		0.001	
Episode Frequency	0.731	0.263	0.001	0.002	0.001	0.001	0.001								
Depression Frequency	0.558	0.239	0.052	0.002	0.079	0.031	0.005	0.011	0.017		0.006				
Mania Frequency	0.557	0.153	0.064	0.001	0.130	0.034	0.003	0.018	0.030		0.010				
Diagnosis			0.003	0.001	0.001	0.002	0.001				0.001	0.002	0.001	0.002	0.068
Predominant Polarity	0.001	0.003	0.148	0.038	0.030	0.013	0.106	0.012	0.012	0.182	0.116	0.002			0.001
Polarity at Onset			0.332	0.005	0.031	0.001	0.032		0.034	0.001	0.027	0.026		0.002	
Psychosis	0.001	0.010	0.007	0.019	0.076	0.353		0.003		0.014					
Suicide Attempt	0.030	0.008	0.010	0.166	0.007	0.005	0.002	0.214	0.037	0.001					
Anxiety	0.022	0.001		0.031	0.031	0.024	0.077	0.054	0.107	0.043			0.004		
OCD	0.002	0.001		0.002	0.001		0.003		0.001			0.001	0.037	0.062	
Substance Use	0.009	0.002		0.234	0.012	0.006	0.131	0.078	0.009	0.004					
ADHD/LD		0.001	0.001	0.001		0.001		0.001	0.003	0.003	0.001	0.003	0.070	0.029	0.002

Note. The squared loadings of variables correspond to the squared correlations and correlation ratios between the variable and principal component, for quantitative and qualitative variables, respectively. Values > 0 are shown. *Abbreviations:* ADHD/LD, attention-deficit hyperactivity disorder and/or learning disability; OCD, obsessive-compulsive disorder; PC, principal component.

Table S4. Variable Coordinates on Principal Components per Sample

Variable	Halifax			NIMH		Combined	
	PC 1	PC 2	PC 3	PC 1	PC 2	PC 1	PC 2
Age of Onset	-0.001	**-0.918**	0.166	-0.052	**-0.931**	-0.042	**-0.926**
Depression Onset	0.141	**-0.833**	-0.054	-0.002	**-0.844**	0.024	**-0.842**
Mania Onset	-0.161	**-0.722**	**-0.478**	-0.092	**-0.830**	-0.112	**-0.824**
Episode Frequency	**0.996**	-0.005	0.041	**0.996**	0.046	**0.996**	0.038
Depression Frequency	**0.956**	-0.022	-0.115	**0.890**	0.003	**0.892**	-0.001
Mania Frequency	**0.947**	0.008	0.158	**0.809**	0.098	**0.838**	0.081
Diagnosis = BD-I	0.038	-0.027	**0.336**	0.003	0.004	0.012	0.009
Diagnosis = BD-II	-0.066	0.047	**-0.577**	-0.032	-0.042	-0.057	-0.043
Predom. Polarity = Balanced	0.101	-0.176	**0.128**	0.062	-0.014	0.072	-0.032
Predom. Polarity = Depressive	-0.091	0.108	**-0.598**	-0.002	-0.007	-0.028	-0.004
Predom. Polarity = Manic	-0.006	0.067	**0.555**	-0.066	0.024	-0.049	0.039
Polarity at Onset = Depression	-0.096	0.003	**-0.527**	0.048	0.030	0.012	0.016
Polarity at Onset = Mania	0.113	-0.004	**0.616**	-0.054	-0.034	-0.014	-0.018
Psychosis = No	0.058	-0.056	-0.277	0.025	-0.117	0.027	-0.115
Psychosis = Yes	-0.053	0.051	0.251	-0.021	0.097	-0.023	0.096
Suicide Attempt = No	-0.088	-0.163	0.099	-0.090	-0.155	-0.088	-0.156
Suicide Attempt = Yes	0.131	0.243	-0.148	0.107	0.183	0.108	0.190
Anxiety Disorder = No	-0.132	-0.198	-0.108	-0.079	-0.076	-0.086	-0.092
Anxiety Disorder = Yes	0.191	0.286	0.157	0.133	0.128	0.142	0.151
OCD = No	0.006	0.003	0.005	-0.018	-0.039	-0.014	-0.034
OCD = Yes	-0.035	-0.017	-0.031	0.069	0.151	0.057	0.138
Substance Use = No	-0.087	0.037	-0.157	-0.039	-0.092	-0.053	-0.078
Substance Use = Yes	0.144	-0.062	0.259	0.044	0.103	0.062	0.092
ADHD/LD = No	0.020	-0.063	-0.002	0.004	-0.020	0.006	-0.026
ADHD/LD = Yes	-0.066	0.207	0.006	-0.016	0.082	-0.025	0.099

Note. Variable coordinates after varimax-rotation. Values in bold are variables with squared loading scores > 0.1, reported in the main text. *Abbreviations:* ADHD/LD, attention-deficit hyperactivity disorder and/or any learning disability; BD-I, bipolar I disorder; BD-II, bipolar II disorder; OCD, obsessive-compulsive disorder; PC, principal component; Predom. polarity, predominant polarity.

Table S5. Mixed Effects Model Results for Overall Similarity Using Euclidean Distance

	**Model: Euclidean Distance ~ Relatedness + (1 | Family ID)**					
**Sample**	**Term**	***β***	**SE**	***β*/SE**	***df***	***p***
**Halifax**	Intercept	1.343	0.049	27.510	336	< 0.001
	Relatedness	-0.312	0.134	-2.328	411	**0.020**
**NIMH**	Intercept	1.057	0.058	18.167	14	< 0.001
	Relatedness	-0.275	0.127	-2.165	17	**0.045**
	**Model: Euclidean Distance ~ Relatedness + Cohort + (1 | Family ID)**					
**Combined**	Intercept	1.029	0.025	40.662	314	< 0.001
	Relatedness	-0.234	0.084	-2.782	25	**0.010**
	Cohort: NIMH	-0.010	0.026	-0.391	11	0.703

Note. Fixed effect significance of $p < 0.05$ in bold.

Table S6. Mixed-effects Model Results for Familiality of Individual Variables

Halifax		Model: Absolute Difference ~ Relatedness + (1 | Family ID)					
	**Variable**	**Term**	***β***	**SE**	***β*/SE**	***p***	**Corrected-*p***
	Age of onset	Intercept	1.02	0.06	18.21	< 0.001	-
		Relatedness	-0.53	0.22	-2.45	0.014	0.074
	Onset depression	Intercept	1.10	0.06	17.18	< 0.001	-
		Relatedness	-0.50	0.22	-2.25	0.025	0.074
	Onset mania	Intercept	1.08	0.05	22.16	< 0.001	-
		Relatedness	-0.01	0.20	-0.07	0.948	0.953
	Frequency of total episodes	Intercept	0.66	0.11	6.06	< 0.001	-
		Relatedness	0.25	0.27	0.95	0.343	0.515
	Frequency of depressions	Intercept	0.76	0.11	6.74	< 0.001	-
		Relatedness	0.02	0.31	0.06	0.953	0.953
	Frequency of manias	Intercept	0.54	0.10	5.42	< 0.001	-
		Relatedness	0.53	0.27	1.98	0.048	0.097
**NIMH**		**Model: Absolute Difference ~ Relatedness + (1 | Family ID)**					
	**Variable**	**Term**	***β***	**SE**	***β*/SE**	***p***	**Corrected-*p***
	Age of onset	Intercept	1.02	0.03	39.86	0.000	-
		Relatedness	-0.24	0.06	-3.75	0.000	**< 0.001**
	Onset depression	Intercept	1.01	0.02	41.84	0.000	-
		Relatedness	-0.11	0.06	-1.81	0.071	0.098
	Onset mania	Intercept	1.06	0.03	41.65	0.000	-
		Relatedness	-0.26	0.06	-4.07	0.000	**< 0.001**
	Frequency of total episodes	Intercept	0.66	0.06	10.99	0.000	-
		Relatedness	-0.30	0.16	-1.84	0.081	0.098
	Frequency of depressions	Intercept	0.70	0.06	11.08	0.000	-
		Relatedness	-0.36	0.10	-3.56	0.001	**0.001**
	Frequency of manias	Intercept	0.54	0.06	9.58	0.000	-
		Relatedness	-0.16	0.19	-0.84	0.417	0.417

Note. Fixed effect significance of corrected-*p* < 0.05 in bold. Corrected-*p* used Benjamini-Hochberg adjustment for false-discovery rate. Absolute difference of relatives/controls and probands for standardized variables.

Table S7. Correlation of Variable Missingness and Squared Loadings per Cohort.

**Cohort**	**PC**	**Pearson's *r***	**95% CI**	***p***	**Corrected-*p***
**Halifax**	**1**	-0.221	[-0.659, 0.329]	0.429	0.715
	**2**	-0.374	[-0.744, 0.172]	0.170	0.561
	**3**	-0.602	[-0.852, -0.130]	0.018	0.149
	**4**	0.323	[-0.227, 0.716]	0.241	0.561
	**5**	-0.150	[-0.615, 0.392]	0.593	0.801
	**6**	0.188	[-0.359, 0.639]	0.501	0.752
	**7**	0.131	[-0.408, 0.603]	0.641	0.801
	**8**	0.309	[-0.241, 0.709]	0.262	0.561
	**9**	-0.326	[-0.718, 0.224]	0.236	0.561
	**10**	-0.285	[-0.696, 0.266]	0.303	0.568
	**11**	-0.051	[-0.549, 0.474]	0.858	0.979
	**12**	-0.348	[-0.730, 0.200]	0.203	0.561
	**13**	0.593	[0.116, 0.848]	0.020	0.149
	**14**	-0.007	[-0.518, 0.507]	0.979	0.979
	**15**	-0.017	[-0.525, 0.500]	0.952	0.979
**NIMH**	**1**	0.047	[-0.477, 0.546]	0.869	0.869
	**2**	-0.106	[-0.586, 0.430]	0.707	0.869
	**3**	0.814	[0.518, 0.936]	< 0.001	**0.003**
	**4**	-0.231	[-0.665, 0.319]	0.407	0.869
	**5**	0.533	[0.029, 0.821]	0.041	0.306
	**6**	0.078	[-0.452, 0.568]	0.782	0.869
	**7**	-0.139	[-0.608, 0.402]	0.621	0.869
	**8**	-0.173	[-0.629, 0.372]	0.538	0.869
	**9**	0.051	[-0.473, 0.549]	0.856	0.869
	**10**	-0.069	[-0.561, 0.460]	0.807	0.869
	**11**	0.159	[-0.385, 0.620]	0.572	0.869
	**12**	0.112	[-0.424, 0.591]	0.690	0.869
	**13**	-0.237	[-0.668, 0.313]	0.395	0.869
	**14**	-0.215	[-0.655, 0.334]	0.442	0.869
	**15**	-0.166	[-0.625, 0.378]	0.554	0.869

Note. Pearson's correlation coefficient for unrotated squared loadings of each principal component and proportion missingness in variables per cohort. Corrected-$p < 0.05$ in bold. Corrected-p used Benjamini-Hochberg adjustment for false-discovery rate. Proportion of missing information calculated using only the probands (i.e. the datasets on which the PCA was performed, prior to projecting relatives onto the space). *Abbreviations:* CI, confidence interval; PC, principal component.

Figure S1. Randomized Control Pairing Methods.

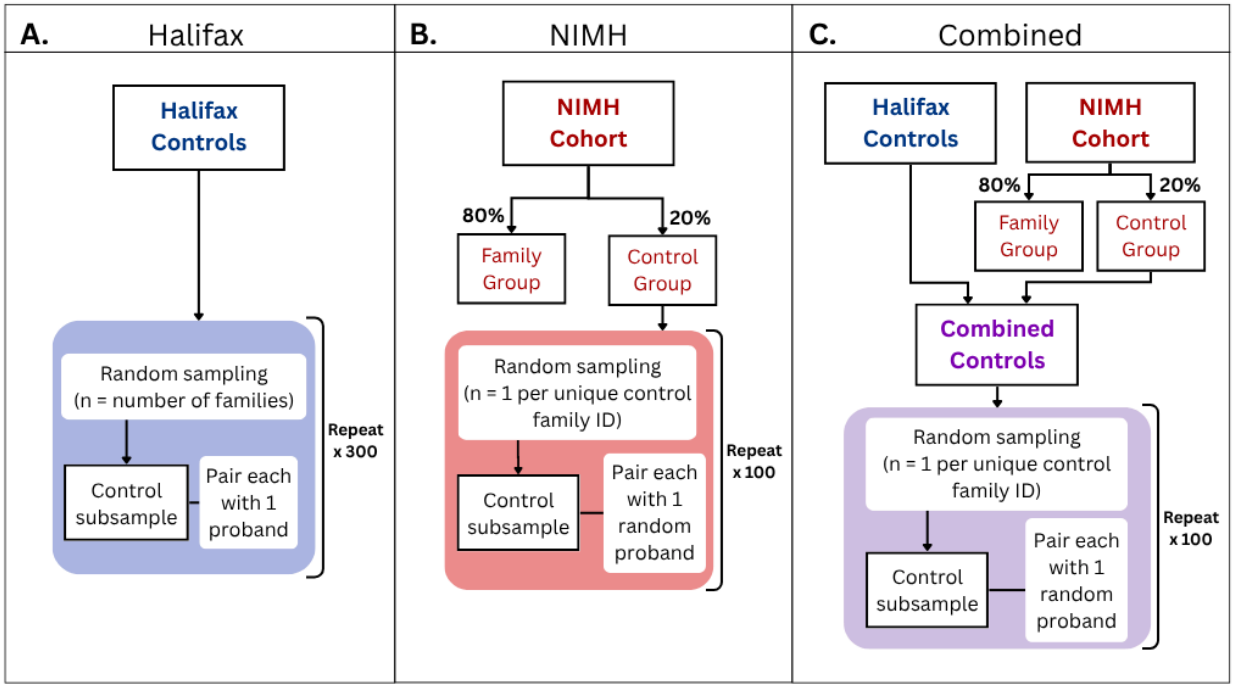

Note. Controls refer to individuals diagnosed with bipolar disorder that are unrelated to subjects in the family group. The Halifax cohort used a control sample that was recruited separately from the family sample, whereas 20% of families in the NIMH cohort were partitioned and designated as the control group.

Figure S2. Percent of Variance Explained by Principal Components per Sample.

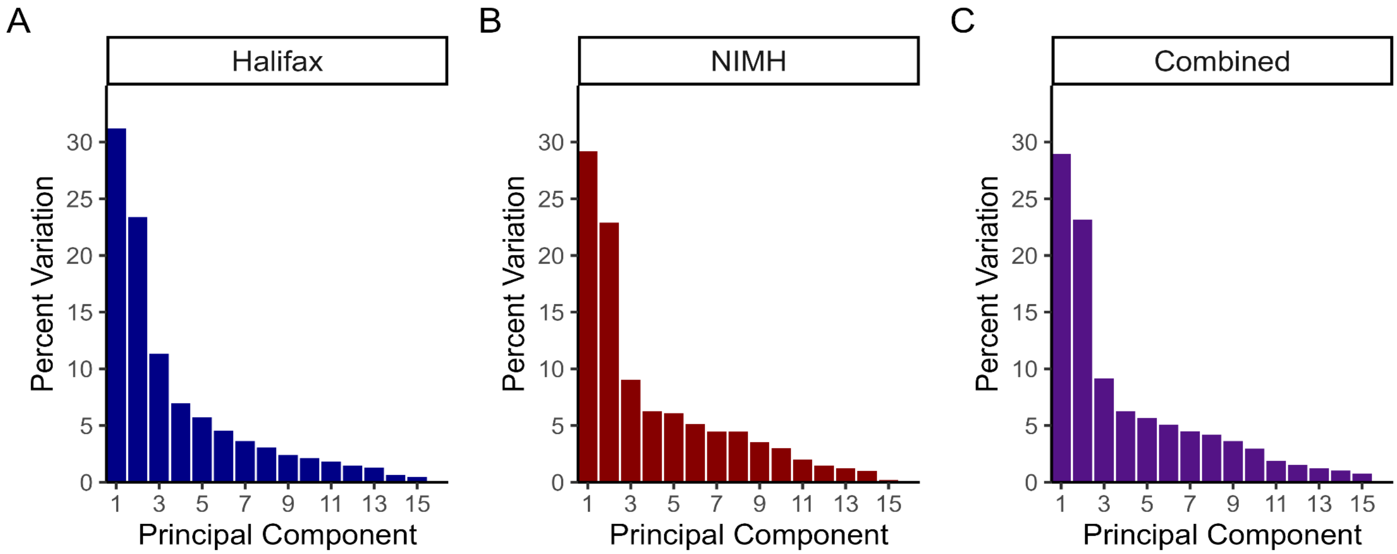

Note. Principal components (PCs) with eigenvalues > 1 were retained for subsequent analyses. (A) PCs 1-3 explained 65.9% of variance in the Halifax dataset; (B) PC 1 and 2 explained 52.1% of variance in the NIMH dataset; and (C) PC 1 and 2 explained 52.1% of variance in the combined samples.

Figure S3. Other Principal Component Biplots for Halifax Cohort

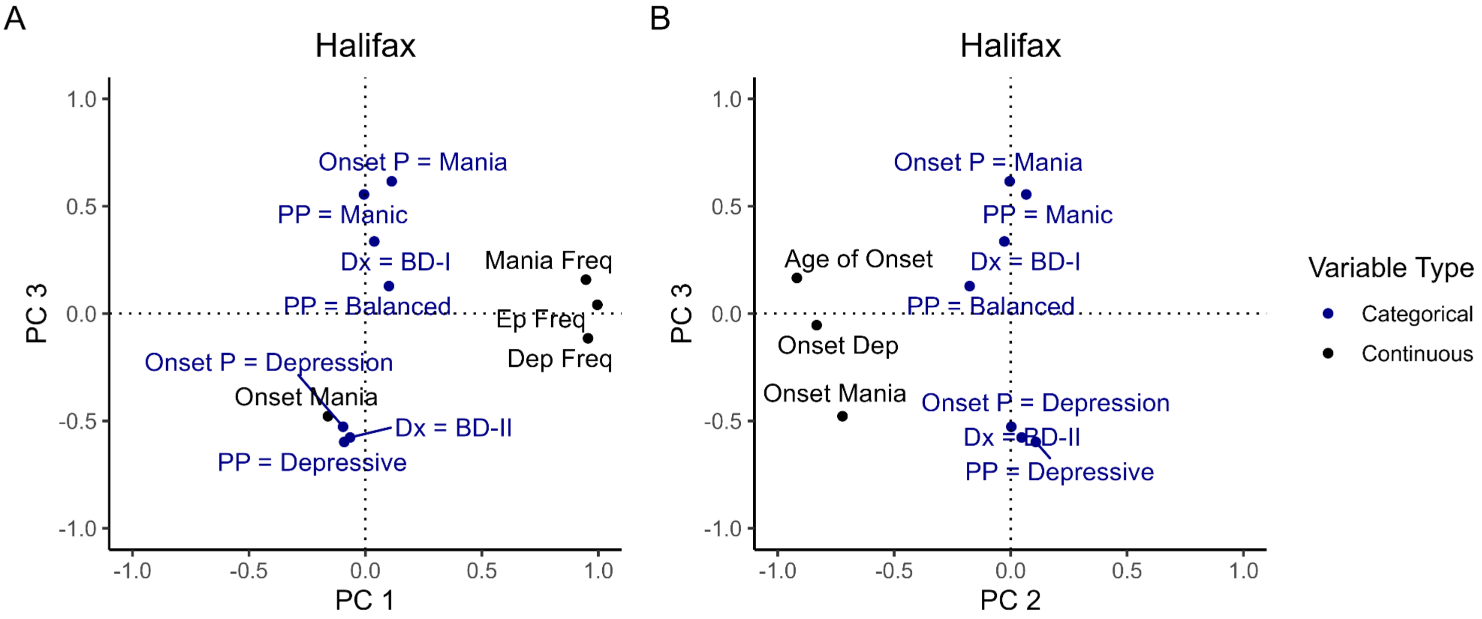

Note. (A) Variable coordinates on principal components 1 and 3, and (B) variable coordinates on principal components 2 and 3 in the Halifax cohort. Only variables with squared loadings > 0.1 after varimax rotation are shown. *Abbreviations*: BD-I, bipolar I disorder; BD-II, bipolar II disorder; Dep, depression; Dx, diagnosis; Ep, episode; Freq, frequency; Onset P, polarity at onset; PC, principal component; PP, predominant polarity.
